# Some Points in the Treatment of Enteric Fever

**Published:** 1910-09-10

**Authors:** A. Knyvett Gordon

**Affiliations:** formerly Medical Superintendent of Monsall Hospital and Lecturer on Infectious Diseases in the University of Manchester.


### Hospital Clinics.

### SOME POINTS IN THE TREATMENT OF ENTERIC FEVER.

By A. KNYVETT GORDON, M.B.Cantab., formerly Medical Superintendent of Monsall Hospital and Lecturer on Infectious Diseases in the University of Manchester.

It has often occurred to me that if one takes the subject of enteric fever as it is treated in most of the modern text-books of medicine, one finds it handled as if no very great change in our knowledge of the pathology of that disease or in our methods of dealing with it at the bedside had taken place in the last few years; in some works, in fact, the article might have been written twenty years ago.

As a matter of fact, however, such change has occurred, and certain discoveries in the field of clinical pathology have been reflected in the practice of those physicians who have had to deal with the disease in bulk, so to speak, with the result not only of considerably reducing the hospital mortality of the disease, but of—even more markedly—alleviating the sufferings of those afflicted with it. I purpose, therefore, mentioning some points which have struck me as essential in the treatment of Enterica, mainly from the point of view of the hospital resident, though, as will be seen, there is no great difficulty in adapting most of them to the exigencies of private practice.

### Changed Views.

Let us first see how our conception of the pathology of the disease has changed. Formerly we regarded the patient as one who was suffering essentially from ulceration of a more or less considerable portion of his intestine, which resulted primarily in diarrhœa, and sometimes was responsible also for intestinal distension, and often for perforation of the bowel, with its necessarily fatal termination. Certain toxins were formed at the site of the ulcers which, when absorbed into the circulation, gave rise to the general symptoms of pyrexia and prostration, and as a necessary consequence of their action on the cardiac muscle the patient suffered a prolonged and debilitating convalescence, which often rendered him incapable of completely performing his ordinary work for many months. Clinically, this view of the disease meant necessarily that the patient was fed on an entirely fluid diet consisting often of milk alone not only during the pyrexial period, but usually for a fortnight or more after the temperature had fallen; every slight rise of temperature after solid or soft food had been commenced was regarded as an indication that the physician had been too venturesome and that a return to the fluid dietary was indicated.

Nowadays, however, we do not regard the ulceration quite as the prime factor in the disease; we know that in the first week of the illness the B. Typhosus is found in the circulating blood in almost every case when a careful technique is employed, and that as the illness progresses the organisms leave the blood and are discharged, *via* the bile, into the duodenum, so that actually more bacilli are present in the commencement of the small intestine than at the site of the ulcers themselves; indeed, it is not unlikely that the ulceration is quite a secondary process and is due to irritation of the intestinal lymphatic patches by the bacilli which have entered from above. We know also that in fatal cases the degree of toxæmia observed at the bedside is not proportional to the extent of ulceration found post-mortem.

### The Modern Idea.

This work of the pathologist was, however, preceded by a change in the method of treating enteric fever which has been practised for many years now in hospitals, though it does not appear to have found its way into private practice any more than into the text-books. It consists essentially in recognising that diarrhœa is not, in the average case, a necessary symptom of enteric fever, but that it can readily be produced by the administration of milk (or perhaps any other food) after the patient has shown his inability to digest it. In practice the diarrhœa almost always ceases when the milk is stopped, and the milk curds, which are an essential feature of the "typical typhoid stool," disappear from the fæces. Moreover, the converse to this also holds good, namely, that the "typhoid stool" is often seen in other diseases (notably in cases of septic scarlet fever) when milk is inadvertently given to excess. The result of this change in the dietary of the enteric patients was not only a great reduction in the immediate mortality (which persisted irrespective of the varying severity of different epidemics), but also in an even more marked diminution in the incidence of those complications—namely, perforation of the intestine and relapse of the disease, which had been previously held to be due to premature resumption of solid food.

The modern practice in this respect may be summed up in the maxim to feed the patient irrespective of his temperature chart on such food as he can digest, the idea being to enable him to combat the toxæmia, which is the essential feature of the complaint, by providing him with as much nutriment as we safely can.

### How it Works.

How does this work out in practice? In the great majority of cases admitted to the fever hospital the patient has diarrhœa and is passing milk-curds in the stools, so as a rule he is given nothing but water for twenty-four or even forty-eight hours; if he is very weak, a little egg albumen and possibly glucose is added to the water. When the diarrhœa has ceased, as it usually does, albumen-water, glucose, and perhaps baked custard and jelly, are administered.

Meantime attention is paid to the toilette of the mouth, for in a large number of cases the dry tongue is a sign of oral sepsis rather than of enteric fever; carious teeth are extracted under local anæsthesia.

In the majority of cases—again irrespective of the temperature chart—the patient is now hungry and has no distension of the abdomen and his tongue is moist. So the diet is gradually extended by the inclusion of bread and milk, bread and butter, pounded fish beaten up, or poached eggs, and ultimately pounded chicken, the essential point being to vary the menu from day to day and to give the patient such a quantity of food as to leave him slightly hungry after each meal. Tea and coffee are given freely and are generally much appreciated by the patient. Shortly, the indications for the use of solid—though carefully pounded—food are hunger, a moist tongue, and absence of diarrhœa and abdominal distension; but there remain, of course, some cases—generally those whom the physician does not see, or perhaps does not correctly diagnose, at a sufficiently early period of the illness—where diarrhœa is profuse and intractable and is accompanied by meteorism.

Here one must give fluid food only, and albumen-water is usually our sheet-anchor, though some preparation of concentrated milk proteid may often be safely and advantageously added; milk itself almost always aggravates the trouble, and beef-tea or meat extracts are quite inadmissible. Drugs, however, are sometimes useful, and I have found enemata containing turpentine very helpful sometimes, and also medical Izal oil given internally in the form of an emulsion in mucilage of tragacanth in doses of three minims of the pure oil (to two ounces of the emulsion) every two hours by the mouth. Personally I do not like opium in these cases, it almost always increases the distension, and thus tends to favour the occurrence of perforation of the intestine, while it completely masks the signs of this catastrophe when it arises. A long, soft tube carefully introduced into the rectum, and allowed to remain there for an hour, is often useful.

### Some Points in Treatment.

The use of Izal oil perhaps demands a word of explanation; it is not given with any idea of disinfecting the ulcers; in the light of modern pathology it probably would not serve any very useful purpose if we could do this, and we fairly obviously cannot, for any antiseptic if soluble would be absorbed before it could reach the ulcerated surface, and if insoluble is in practice passed unchanged per rectum; but the Izal emulsion is probably absorbed in or near the duodenum, and may perhaps check the formation of toxins which irritate the intestine lower down. Whatever theory, however, be held as to its *modus operandi*, I have no doubt from a fairly extensive trial that it checks diarrhœa.

It is also a powerful diuretic and diaphoretic, and this brings me to the next point—namely, whether we can assist in the elimination of toxins through the skin and kidneys. The first suggestion for this purpose was the routine use of the cool bath, and there can be no doubt that when the external temperature is high, as in Australia and America, whence come the most markedly favourable reports of its use, it may be employed almost as a routine measure. But otherwise, that is to say during the greater part of the year in this country, I believe that cold bathing is best reserved for the sthenic type of case. In robust adults, with full pulses and much delirium (of the violent, as distinguished from the low muttering type), it often acts like a charm; but I have seen some untoward results from its use in feeble patients and in those suffering from diarrhœa. As an antipyretic, cold bathing or sponging is sometimes efficacious, but it is seldom necessary or advisable to treat the pyrexia *per se*—one should prescribe for the patient and not for his temperature chart. It would seem that the enthusiasm for routine cold bathing has rather abated of late years.

A much more efficient measure, in my experience, lies in the administration of large quantities of water, or flavoured barley water, by mouth, and a skilful nurse will often get her patient to drink from five to six pints of fluid in the twenty-four hours, this form of treatment being most efficacious when combined with diuretics such as "imperial drink" or the aforesaid Izal emulsion.

In the treatment of the ordinary case we have, then, three indications to fulfil—namely, to keep up the patient's strength by the administration of adequate nourishment in order that his leucocytes may be in a condition to afford an efficient defence against the attacks of the bacillus or its toxins, to keep the intestine as still as possible by the avoidance of anything tending to cause diarrhœa, and to assist as far as we are able in the elimination of toxins through the skin and kidneys.

### Complications.

A few words may now be said about the treatment of certain complications of the disease, and we will take first the occurrence of heart failure. This may be either sudden or gradual, and in the former case is almost invariably accompanied by extreme dilatation of the left ventricle, which can be detected by light percussion; it is particularly liable to occur in the course of attacks which are characterised by nervous prostration rather than by much pyrexia, the type of disease, in fact, which we often meet with either in thin, worried-looking men or in overworked women. In many cases the administration of stimulants by the mouth is useless as they are not absorbed sufficiently quickly, and hypodermic injections are but little, if at all, more efficacious, as the capillary circulation is in a state of stasis. Very many patients, in fact, die immediately, but I have seen not a few saved by the timely application by the nurse of a towel or bath sponge wrung out of very hot water to the præcordia, and it is as well that instructions to this effect should be left with the attendants. Inasmuch as the condition is invariably accompanied by extreme pallor of the face, its occurrence can be detected by a careful nurse, or even by the patient's relatives. When the emergency has been treated a relapse must be guarded against by the hypodermic administration of strychnine at regular intervals.

Failing Heart and Hæmorrhage.

The treatment of the gradually failing heart is more difficult, as in many cases it is undoubtedly due to a specific action of the typhoid toxin on the cardiac muscle, and in consequence stimulants are not of much avail, and I am convinced that the administration of digitalis—apart from pre-existing valvular disease—is not only erroneous but dangerous, and there is, moreover, considerable risk of the dose of the drug being increased under the belief that it is not acting sufficiently while the patient is in reality suffering from digitalis poisoning.

It is necessary here to mention the use of alcohol in enteric fever. My own view is that, in large doses, given for rapidly occurring heart-failure, and discontinued as soon as the emergency is past, brandy is very useful indeed, but that the common practice of administering two or three ounces of spirit daily for two or three weeks is most harmful. I have never satisfied myself that such doses were of any value to the circulation, and I am sure that they often produce or aggravate diarrhœa. Probably, strychnine is the most valuable drug we possess as far as the heart is concerned, and, if given hypodermically, does not seem to increase intestinal peristalsis.

The treatment of hæmorrhage from the intestine not only resolves itself into the administration of opium very freely, but it is also necessary to avoid the use of any hæmostatic which has the effect of raising the general blood-pressure. What then happens is that the action on the vaso-motor system outweighs the local effect on the injured vessel, and in practice such drugs as adrenalin, ergot, gallic acid, and the like almost always increase the hæmorrhage.

Just as it is necessary to use opium in hæmorrhage, so it is essential to avoid it in anything that may possibly turn out to be perforation of the intestine. The key to the successful treatment of the latter complication is that the surgeon shall have the opportunity of opening the abdomen in the pre-peritonitic stage, and this is impossible if opium be given. Moderately severe abdominal pain in enteric fever is far more often due to perforation than is usually supposed, and the result of the advice given in most of the text-books to give opium for abdominal pain is accountable for the descriptions in the same volumes of the symptoms of perforation, which are in reality those of the subsequent purulent peritoneal effusion. If the patient has an attack of colic, it can usually be relieved by the application of a hot fomentation, and, indeed, often by a cup of hot tea. In practice patients do not always, or, as I believe, usually suffer from severe collapse when perforation occurs. It is not necessary to consider seriously the view that any other procedure except prompt laparotomy can be of any avail in the treatment of this distressing occurrence.

